# Super enhancer related gene ANP32B promotes the proliferation of acute myeloid leukemia by enhancing MYC through histone acetylation

**DOI:** 10.1186/s12935-024-03271-y

**Published:** 2024-02-22

**Authors:** Xiaomei Wan, Jianwei Wang, Fang Fang, Yixin Hu, Zimu Zhang, Yanfang Tao, Yongping Zhang, Juanjuan Yu, Yumeng Wu, Bi Zhou, Hongli Yin, Li Ma, Xiaolu Li, Ran Zhuo, Wei Cheng, Shuqi Zhang, Jian Pan, Jun Lu, Shaoyan Hu

**Affiliations:** 1 Children’s Hospital of Soochow University, Suzhou, 215003 China; 2 Department of Pediatrics, The First Affiliated Hospital of Wannan Medical College, Wuhu, 24100 China; 3 Institute of Pediatric Research, Children’s Hospital of Soochow University, No.92 Zhongnan Street, SIP, Suzhou, 215003 China; 4 Department of Pediatrics, The First Affiliated Hospital of Bengbu Medical College, Bengbu, 233004 China; 5 Suzhou Hospital of AnHui Medical University, Suzhou, 234000 China; 6 Department of Hematology, Children’s Hospital of Soochow University, No.92 Zhongnan Street, SIP, Suzhou, 215003 Jiangsu China

**Keywords:** Acute myeloid leukemia, ANP32B, C-MYC, Histone Acetylation, H3K27ac

## Abstract

**Background:**

Acute myeloid leukemia (AML) is a malignancy of the hematopoietic system, and childhood AML accounts for about 20% of pediatric leukemia. ANP32B, an important nuclear protein associated with proliferation, has been found to regulate hematopoiesis and CML leukemogenesis by inhibiting p53 activity. However, recent study suggests that ANP32B exerts a suppressive effect on B-cell acute lymphoblastic leukemia (ALL) in mice by activating PU.1. Nevertheless, the precise underlying mechanism of ANP32B in AML remains elusive.

**Results:**

Super enhancer related gene ANP32B was significantly upregulated in AML patients. The expression of ANP32B exhibited a negative correlation with overall survival. Knocking down ANP32B suppressed the proliferation of AML cell lines MV4-11 and Kasumi-1, along with downregulation of C-MYC expression. Additionally, it led to a significant decrease in H3K27ac levels in AML cell lines. In vivo experiments further demonstrated that ANP32B knockdown effectively inhibited tumor growth.

**Conclusions:**

ANP32B plays a significant role in promoting tumor proliferation in AML. The downregulation of ANP32B induces cell cycle arrest and promotes apoptosis in AML cell lines. Mechanistic analysis suggests that ANP32B may epigenetically regulate the expression of MYC through histone H3K27 acetylation. ANP32B could serve as a prognostic biomarker and potential therapeutic target for AML patients.

**Supplementary Information:**

The online version contains supplementary material available at 10.1186/s12935-024-03271-y.

## Introduction

Acute myeloid leukemia (AML) is a malignant hematological tumor caused by genetic alterations and epigenetic disorders in myeloid precursor cells [1]. In the pediatric population, AML constitutes 20% of leukemias [2, 3]. Despite the overall survival rate of 75% in pediatric AML, the prognosis remains unfavorable for patients with relapsed or refractory disease [4]. Approximately 30% of children diagnosed with de novo AML undergo relapse, posing a substantial challenge in achieving a cure in the majority of cases [5]. Consequently, there is an urgent demand for targeted pharmaceutical agents and refined therapeutic approaches to enhance outcomes and extend the survival duration for pediatric AML patients.

Super-enhancers (SEs) is refers to a substantial aggregation of transcriptionally active enhancers. The identification of SEs can be achieved through active histone markers such as H3K27ac, H3K4me1, and transcription cofactor p300 [6]. SEs HOX, GFI1b [7, 8] have been shown to be involved in the development of AML, suggesting that SEs stimulate the activation of oncogenes, thereby initiating the development of AML. However, the super enhancer spectrum in children’s AML is unclear.

Therefore, we collected bone marrow samples from 11 pediatric AML patients to detect H3K27ac modification signals in tumor cells by chromatin immunocoprecipitations (ChIP-Seq). Additionally, we included B-ALL bone marrow samples as control samples to screen for SEs specific to pediatric AML. We screened 200 genes specifically modified by H3K27ac in pediatric AML, including ANP32A and ANP32B. The role of ANP32A [9] in the pathogenesis of leukemia has been substantiated by studies. Given its membership within the acidic leucine-rich nuclear phosphoprotein 32 kDa (ANP32) protein family [10], The involvement of the ANP32B gene in AML has piqued our keen interest. Notably, ANP32B’s LRR region exhibits binding activity with core histone H3-H4. The concave parallel β folding structure of the leucine-rich repeat (LRR) protein mediates the interaction between ANP32B and histones [11]. Conversely, the LCAR region of ANP32B functions as an inhibitor of histone acetylation. ANP32B acts as a histone chaperone, binding to transcription factors and regulating the activity of the Kruppel-like factor 5 (KLF5) transcription factor, thereby specifically inhibiting the transcription of downstream genes [12]. In the context of solid tumors, ANP32B plays a role in promoting liver cancer through the apoptosis pathway and protein interactions in the MARK and PI3K-AKT pathways [13].

The anti-apoptotic protein ANP32B has been shown in multiple studies to function as a direct substrate of caspase-3. Following its down-regulation and subsequent activation of caspase-3, ANP32B can be degraded by the activated caspase-3, thereby inducing cell apoptosis [14]. Additionally, ANP32B has been found to diminish the differentiation ability of leukemia cells [15]. Furthermore, ANP32B has been identified as an inhibitor of p53 activity, playing a crucial role in the regulation of both normal and CML stem cell maintenance [16]. However, a recent investigation has revealed that ANP32B exerts a suppressive effect on B-ALL in mice by activating PU.1 [17]. The findings imply that ANP32B potentially plays a multifaceted role in leukemia.

However, the precise underlying mechanism of ANP32B in the pathogenesis of hematologic malignancies remains elusive. Our previous investigation demonstrated that MZ1, a novel BET inhibitor, elicited significant downregulation of ANP32B genes in AML cell lines [18]. In this study, we observed an upregulation of ANP32B in AML patients, and high expression levels of ANP32B were associated with poor prognosis. The proliferation of AML cells MV4-11 and Kasumi-1 was suppressed by ANP32B knockdown by triggering apoptosis and inhibiting the cell cycle. Furthermore, the knockdown of ANP32B in AML cells exhibited a remarkable inhibition of tumor growth in vivo. Additional analysis indicated a positive correlation between ANP32B and C-MYC expression. Moreover, the downregulation of C-MYC expression in AML cell lines was observed upon the knockdown of ANP32B, suggesting that C-MYC is a downstream target of ANP32B. ANP32B knockdown led to a significant reduction in the levels of acetylated lysine 27 on histone 3 (H3K27ac).

In conclusion, our experimental findings suggest that ANP32B can be considered as a promising biomarker for prognostic assessment and a potential therapeutic target for pediatric AML.

## Materials and methods

### Cell culture

The MV4-11, Kasumi-1 human AML cell lines were obtained from the Chinese Academy of Sciences Cell Bank, and authenticated through Short Tandem Repeat (STR) analysis in 2023. Cells were cultured in RPMI1640 medium (22400089, Gibco, USA) supplemented with 10% fetal bovine serum (Biological Industries, CT, USA), and 1% penicillin–streptomycin (Millipore Sigma, MA, USA), cultured in a humidified incubator at 37 ℃ containing 5% CO_2_, and routinely tested for mycoplasma.

### ChIP-Seq data collection and analysis

In the present study, the raw data of our previous ChIP-Seq datasets for AML samples (GSE188605) were aligned to UCSC hg38 (the reference genome) using Bowtie2 (v 2.3.5) [19], with the following parameters -p 4 -q -x. Peaks were identified using MACS2 (v2.0.9) [20], applying the parameters -g hs -n test -B -q 0.01. The bigwig files of these datasets were then visualized using Integrative Genomics Viewer (IGV) [21] and WashU tool (http://epigenomegateway.wustl.edu/browser/).

### Public Hi-C data collection and analysis

The Hi-C data for THP-1 cell line (GSE126979) was obtained from the Gene Expression Omnibus database. Read mapping and loop calling were performed using HiC-pro (v.3.1.0) [22]. MboI restriction sites in the hg38 build were used for alignment with Bowtie2 as the mapping tool, specifying global options of –very-sensitive -L 30 –score-min L, − 0.6, − 0.2 –end-to-end –reorder and local options of –very-sensitive -L 20 –score-min L, − 0.6, − 0.2 –end-to-end –reorder during mapping procedure with 'GATCGATC' as the ligation site. The results were visualized and graphed using WashU tool (http://epigenomegateway.wustl.edu/browser/).

### White slice assay

MV4-11 and Kasumi-1 cells stably transfected with sh-NC and sh-ANP32B were inoculated into T25 culture bottles for culture. On day 3/5/7 after purinomycin screening, the number and morphology of the knocked down cells were observed under the microscope, and white light microscopic images were taken by the light microscope.

### Cell proliferation assay

Cells (2 × 10^3^/well) were seeded into 96-well plates with 1 µg/ml puromycin (ST551, Beyotime, China) added into the cultural medium. After 48 h and every 2 days, 20 µl CCK-8 solution (Dojindo Molecular Technologies, Tokyo, Japan) was supplemented and incubated for another 2 h. The absorbance at a wavelength of 450 nm was subsequently measured using an A Microplate Absorbance Reader (Thermo, USA).

### Soft agar clone formation analysis

1.2% agarose gel was used to prepare the underlying gel. After curing at room temperature, 2 × 103/ml with 100 µl of cells suspension AAA cell suspensions were added into 0.7% agarose gel to prepare the upper gel. Put six-well plate of soft agar in CO2 incubator at 37 ℃. After a 2-week incubation period, the cells were initially fixed with 100% methanol for a duration of 15 min, followed by staining with Giemsa solution for an hour. Subsequently, colonies were meticulously observed and quantified using an optical microscope (Leica).

### Preparation and infection of lentivirus

As previously described [18], The backbone plasmids Sh-ANP32B (IGE Biotechnology LTD, Guangzhou, China) were co-transfected into 293FT cells along with packaging plasmids psPAX2 and pMD2G (pMD2.G: Cat. No. 12259; psPAX2: Cat. No. 12260; Cambridge, MA, USA). Screen stable cell lines with puromycin (Sigma-Aldrich).

The sequences of shRNA used were as follows:

ANP32B-shRNA-1:

5ʹ-CCGGGAAGAATTTGGACTTGATGAACTCGAGTTCATCAAGTCCAAATTCTTCTTTTTGAATT-3ʹ

ANP32B-shRNA-2:

5ʹ-CCGGGAGGGCTTAACAGCTGAATTTCTCGAGAAATT­CAGCTGTTAAGCCCTCTTTT TGAATT-3ʹ

ANP32B-shRNA-3:

5ʹ-CCGGGCTTACCTACTTGGATGGCTACTCGAGTAGCCATCCAAGTAGGTAAGCTTTTT GAATT-3ʹ

### RNA preparation and real-time PCR expression analysis

According to the manufacturer’s protocol, the cells were subjected to RNA extraction using TRIzol^®^ reagents (Invitrogen, CA, USA). Subsequently, the extracted total RNA was reverse transcribed into cDNA using a high-capacity cDNA reverse transcription kit (Applied Biosystems, CA, USA). For PCR amplification, the reaction system was prepared with LightCycler^®^480 SYBR Green I Master mixture (cat. 04707516001; Roche, Penzberg, Germany), and real-time PCR was performed on the LightCycler 480 system (Roche). The primer sequences are listed in Additional file 1: Table S1.

### Cell cycle analysis

Following the manufacturer's protocol, cells were collected and then fixed overnight in a – 20 ℃ refrigerator after the addition of pre-cooled 75% ethanol. On the following day, the cells were washed with cold phosphate buffered saline (PBS) and treated with PI dye and RNase A (cat. No. 550825; BD Pharmingen^™^, San Diego, CA, USA),. Subsequently, they were incubated at 4 ℃ for 30 min in darkness. The cell cycle was analyzed using Beckman Gallios™ flow cytometry (Beckman, Krefeld, Germany) following standard procedures, and the results were analyzed using FlowJo_V10.

### Cell apoptosis analysis

Follow the manufacturer’s instructions. 1 × 106 cells were collected, washed with pre-cooled 1xPBS, and then resuspended in 100 µl of 1 × binding buffer. Subsequently, fluorescein isothiocyanate (FITC)-AnnexinV (5 µl) and PI (5 µl) were sequentially added (cat. No. 556420; BD Biosciences, Franklin Lakes, NJ, USA). The mixture was incubated away from light for 15 min with an additional volume of 400 µl of 1 × binding buffer. Apoptosis analysis was performed using Beckman Gallios^™^ flow cytometry (Beckman, Krefeld, Germany) following standard procedures, and the results were analyzed using FlowJo_V10.

### Western blotting analysis

The following antibodies should be utilized for Western blot analysis. ANP32B (cat. No. ab200836; Abcam, USA), ANP32A (cat. No. 15810-1-AP; Proteintech, USA) and C-MYC (cat. No. 9402S; Cell Signal Technology, USA), PLK1 (cat. No. ab17056; Abcam, USA), Anti-acetyl-Histone H3 antibody** (**cat. No. 06-599; Millipore, USA**)**, Histone H3 (D1H2) XP^®^ Rabbit mAb (cat. No. 4499S; Cell Signal Technology, USA), H3K27ac (cat. No. ab4729; Abcam, USA), Monoclonal ANTI-FLAG M2 antibody (Lot#SLBS3530V; Sigma, GER), CCNB1(cat. No. ab32053; Abcam, USA), CDK2 (cat. No. ab32147; Abcam, USA), BAX (cat. No. ab32503; Abcam, USA). The reference protein used was GAPDH (cat. No. MA3374; Millipore).

### In vivo experiments

4–6 week-old NSG mice (Shanghai Model Organizations, China) were randomly allocated into sh-NC group and sh-ANP32B group. Each mouse was injected with 2 × 10^6^ AML cells expressing firefly luciferase through the tail vein. Bioluminescence signal values of mice in each group were monitored intermittently by small animal imaging (Berthold, Germany). Liver, spleen and bone were stained with immunohistochemistry and eosin (HE). Antibodies against Ki67(GB111499-100, Servicebio, China), C-MYC (GB13076-50, Servicebio, China), ANP32B (cat. No. ab200836; Abcam, USA) were employed in accordance with the manufacturer's instructions. The Animal Care and Use Committee at the Children's Hospital of Soochow University (CAM-SU-AP#:JP-2018-1) granted approval and licensing for all animal studies.

### RNA sequencing and data analysis

The RNA-seq experiment was conducted following the recommended protocol provided by Novogene Bioinformatics Technology Co., Ltd. (Beijing, China). Collect the cell suspension containing one million cells and discard the supernatant after centrifugation. The cell pellet was preserved in 1 ml Trizol reagent, and stored at – 80 ℃ for RNA-seq sequencing at Novogene in Tianjin. The RNA-seq reads were aligned to the hg38 reference genome using HISAT2 (v2.0.5). Differentially expressed genes were identified through DESeq2 analysis, (p < 0.05 and |log twofold change |> 0.5). The RNA-seq data from this study has been deposited in the GEO database (https://www.ncbi.nlm.nih.gov/geo) under accession code GSE242850.

### Cleavage under targets and tagmentation (CUT&Tag) assay

According to the manufacturer’s instructions, we performed the CUT&Tag Assay using the Hyperactive Universal Cut and Mark Analysis Kit (TD903-01, Vazyme,). Samples incubated overnight with H3K27ac antibody (Item No: AB4729, 2ug, Abcam, Cambridge, UK). The samples were then amplified using the TruePrep Index Kit V2 for Illumina (#TD202) and sent to Tianjin Novogene for sequencing. For CUT&Tag analysis, reads were aligned with hg38 using Bowtie2 (v2.2.5) and then PCR repeat reads were removed using Picard. The replication was merged using samtools merge (v1.15.1). Use MACS2 (v2.2.7) to call the peak based on the parameter -Q 0.01—bdg—SPMR.

### Statistical analysis

All experiments were independently performed at least three times. All statistical analyses were performed using GraphPad PRISM 8.0.2 software (GraphPad Software Inc., La Jolla, CA, USA). The two groups were compared by double-tailed unpaired Student's t test for data analysis. A p value < 0.05 was considered statistically significant (*p < 0.05, **p < 0.01, ***p < 0.001, ****p < 0.0001), while NS indicated no significance."

## Results

### ANP32B is upregulated in AML and associated with poor prognosis in AML patients

**Fig. 1:**
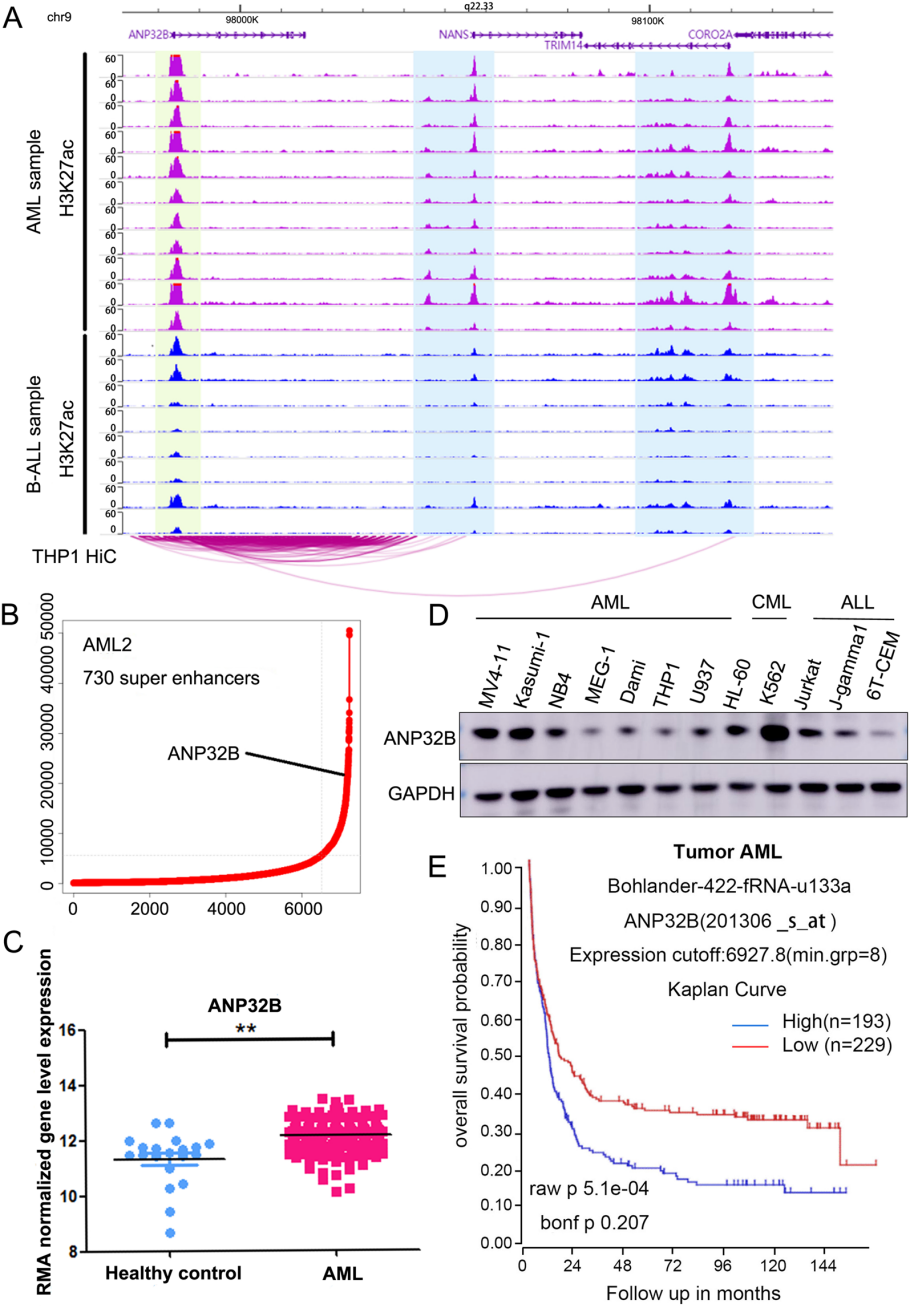
ANP32B is upregulated in AML and correlated with poor clinical characteristics. **A** The ChIP-Seq data demonstrates H3K27ac signaling of ANP32B in AML and B-ALL clinical samples, while Hi-C data analysis reveals interactions between super-enhancer regions and the promoter of ANP32B in AML cells. **B** Super-enhancer profiling in AML sample. Enhancers were ranked by increasing H3K27ac signal in AML sample. Number of super-enhancers identified in AML sample is shown. ANP32B which is associated with super-enhancer in AML sample is shown. **C** ANP32B was highly expressed in AML compared to healthy control according to a public transcriptomic dataset (GSE114868). **D** Western blot assay for ANP32B protein expression in leukemia cell lines. **E** The Kaplan–Meier survival curve from the R2 database (https://hgserver1.amc.nl/cgi-bin/r2/main.cgi)

Through the analysis of ChIP-Seq data obtained from AML and B-ALL samples (Fig. 1A), we observed that the upstream region of ANP32B gene in AML samples exhibits concurrent H3K27ac signals, which were relatively scarce in B-ALL samples. These findings suggested a potential involvement of the enhancer region in the transcriptional regulation of ANP32B. Furthermore, Hi-C data analysis revealed interactions between super-enhancer regions and the promoter of ANP32B in AML samples (track 1–11), indicating their association with the gene loci of ANP32B. Meanwhile we carried out ROSE analysis and added the rank of super-enhancers and the rank of ANP32B enhancer in Additional file 1: Table S2. We also show a representative ranking plot with label of ANP32B enhancer rank from ROSE analysis (Fig. 1B). Targeting BRD4, an important component of the super-enhancer, led to downregulation of ANP32B (Additional file 1: Fig.S1). Consistent with these findings, we conducted a comparative analysis of ANP32B expression patterns between individuals with acute myeloid leukemia (AML) and healthy controls using publicly available transcriptomic data (GSE114868) [23]. Our results revealed a significant upregulation of ANP32B in AML samples (Fig. 1C). Additionally, we conducted western blotting analysis to evaluate the expression levels of ANP32B in leukemia cell lines. Our results revealed differential expression of ANP32B in CML, AML and ALL cell lines (Fig. 1D). Notably, previous studies have reported on the role of ANP32B in CML [16] and ALL [17], suggesting its general significance in leukemia. We focused on the role of ANP32B in AML. The Kaplan–Meier survival curve from the R2 database (https://hgserver1.amc.nl/cgi-bin/r2/main.cgi) indicate a lower expected overall survival rate for individuals with high ANP32B expression in acute myeloid leukemia (AML) compared to those with low ANP32B expression (Fig. 1E). These findings collectively suggest that ANP32B is likely activated through its distal enhancer and that abnormal ANP32B expression is closely associated with AML.

### Altered ANP32B expression affects the viability of AML Cell

**Fig. 2:**
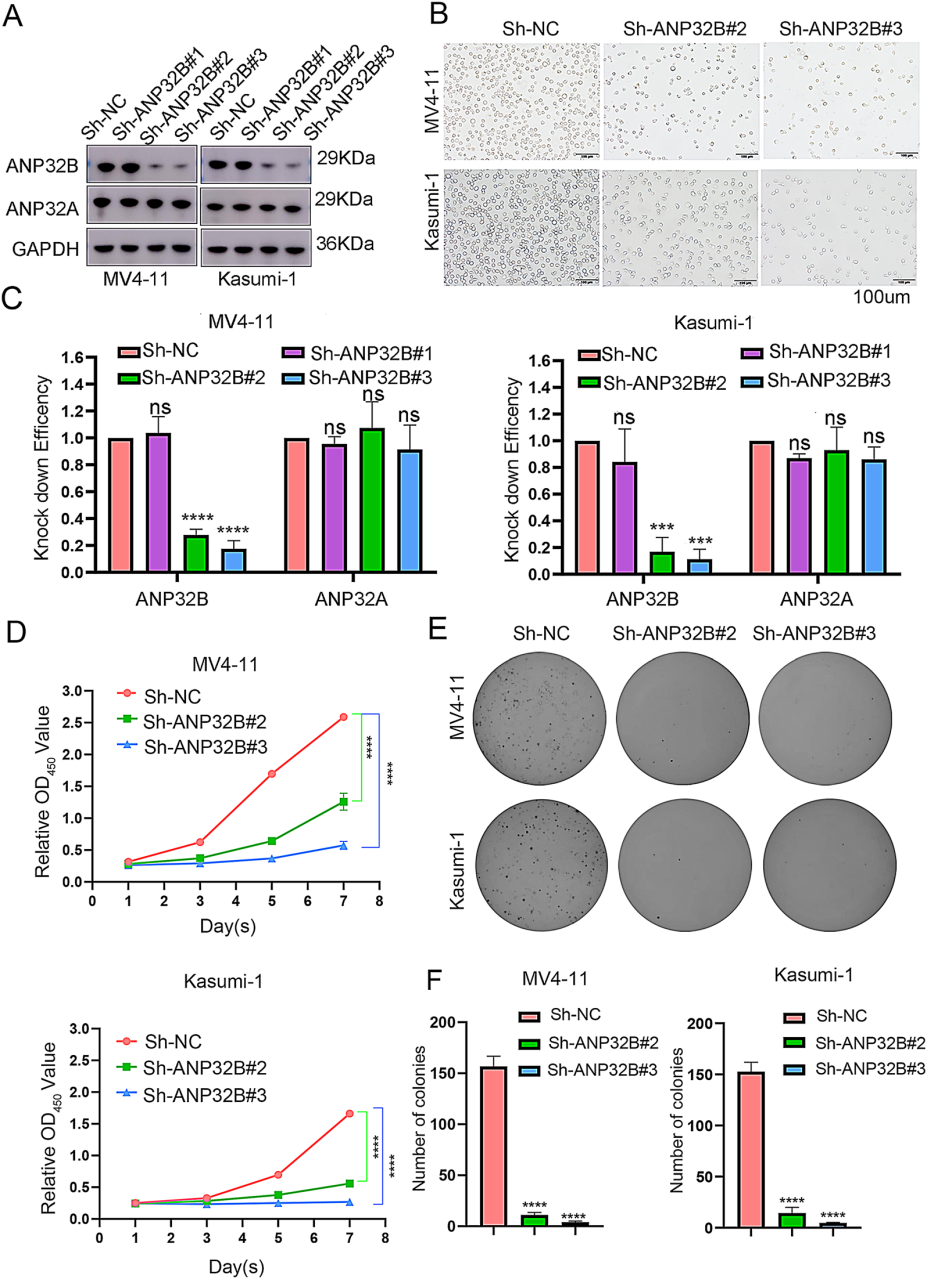
Knockdown of ANP32B inhibit the growth of AML cell lines. **A** Western blotting analysis showed that ANP32B, ANP32A and GAPDH protein pression in MV4-11 and Kasumi-1 cells after ANP32B knockdown. **B** White slice showed that ANP32B knockdown significantly inhibited AML cells proliferation. **C** The knockdown levels of ANP32B and ANP32A in cells was verified by WB. **D** CCK-8 assay detected the proliferation rate of MV4-11 and Kasumi-1 cells after ANP32B knockdown. **E**, **F** Colony-forming assay for MV4-11 and Kasumi-1 cells infected with sh-NC or Sh-ANP32B#2, shANP32B#3

In order to examine the role of ANP32B in AML, we conducted transfection experiments using three ANP32B-targeting short hairpin RNAs (sh-ANP32B#1, sh-ANP32B#2 and sh-ANP32B#3), as well as control sh-NC, in two AML cell lines, MV4-11 and Kasumi-1. Through Western blot and RT-PCR analyses, it was observed that the expression of ANP32B in the cells transfected with ANP32B shRNAs was significantly diminished at the protein and RNA levels, in comparison to the control group (Fig. 2A, C, Additional file 1: Fig. S2). However, the expression of ANP32A remained unaffected (Fig. 2A). Furthermore, the results from the white slice assay demonstrated that the knockdown of ANP32B substantially impeded the proliferation of AML cells, when compared to the control (Fig. 2B). The findings from the CCK-8 assay indicate a significant inhibition of cell proliferation in the ANP32B knockdown group (Fig. 2D). The assessment of tumorigenicity in malignant cells is commonly conducted by evaluating their ability to form colonies in soft AGAR. The results demonstrate a significant reduction in the colony-forming ability of AML cells upon ANP32B knockdown (Fig. 2E and F). Collectively, these results strongly suggest that the abnormal expression of ANP32B plays a crucial role in the viability of AML cells.

### Knockdown of ANP32B enhanced apoptosis in AML cell lines and suppressed cell cycle progression

**Fig. 3:**
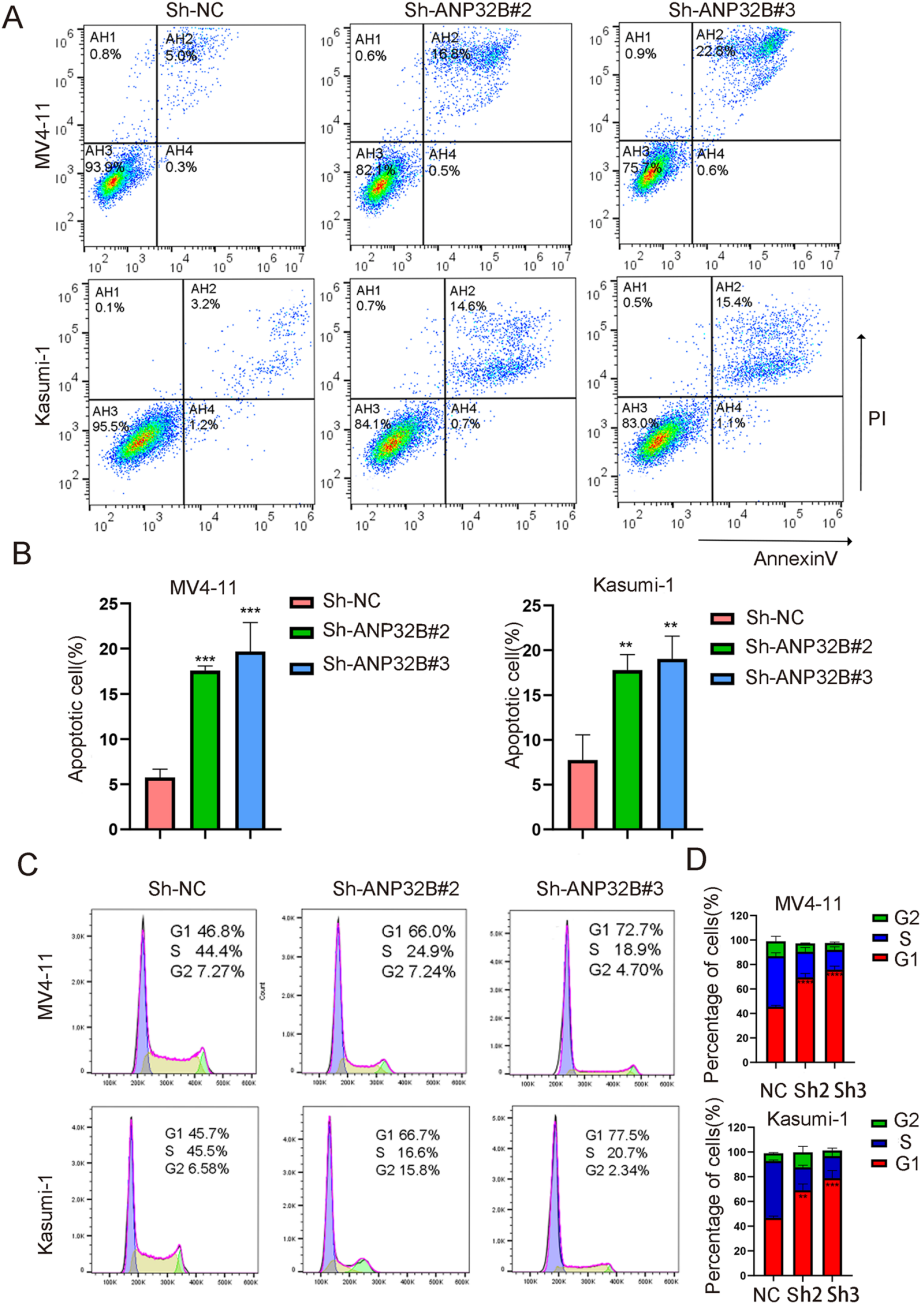
ANP32B knockdown promotes apoptosis and blocks cells cycle. **A**, **B** Flow cytometry using Annexin V staining showed that knockdown of ANP32B increased the apoptotic rates of MV4-11 and Kasumi-1 cell lines. **C**, **D** Knockdown of ANP32B caused cell cycle arrest at G0/G1 phase in MV4-11 and Kasumi-1 cell lines

To investigate the influence of ANP32B expression on apoptosis and cell cycle, we initially conducted flow cytometry analysis utilizing Annexin V and PI to evaluate apoptosis subsequent to ANP32B knockdown in two AML cell lines. The results revealed a significant increase in apoptosis following ANP32B knockdown (Fig. 3A). The quantitative representation of apoptotic cells is depicted in Fig. 3B. Additionally, ANP32B knockdown led to cell cycle arrest in the G0/G1 phase, with a noticeable decrease in the G2/M and S phases compared to scramble cells (Fig. 3C, and D). Collectively, these outcomes indicate that depletion of ANP32B resulted in cell cycle arrest and promoted apoptosis in AML cells.

### Knockdown of ANP32B inhibited viability of AML cells in Vivo

**Fig. 4:**
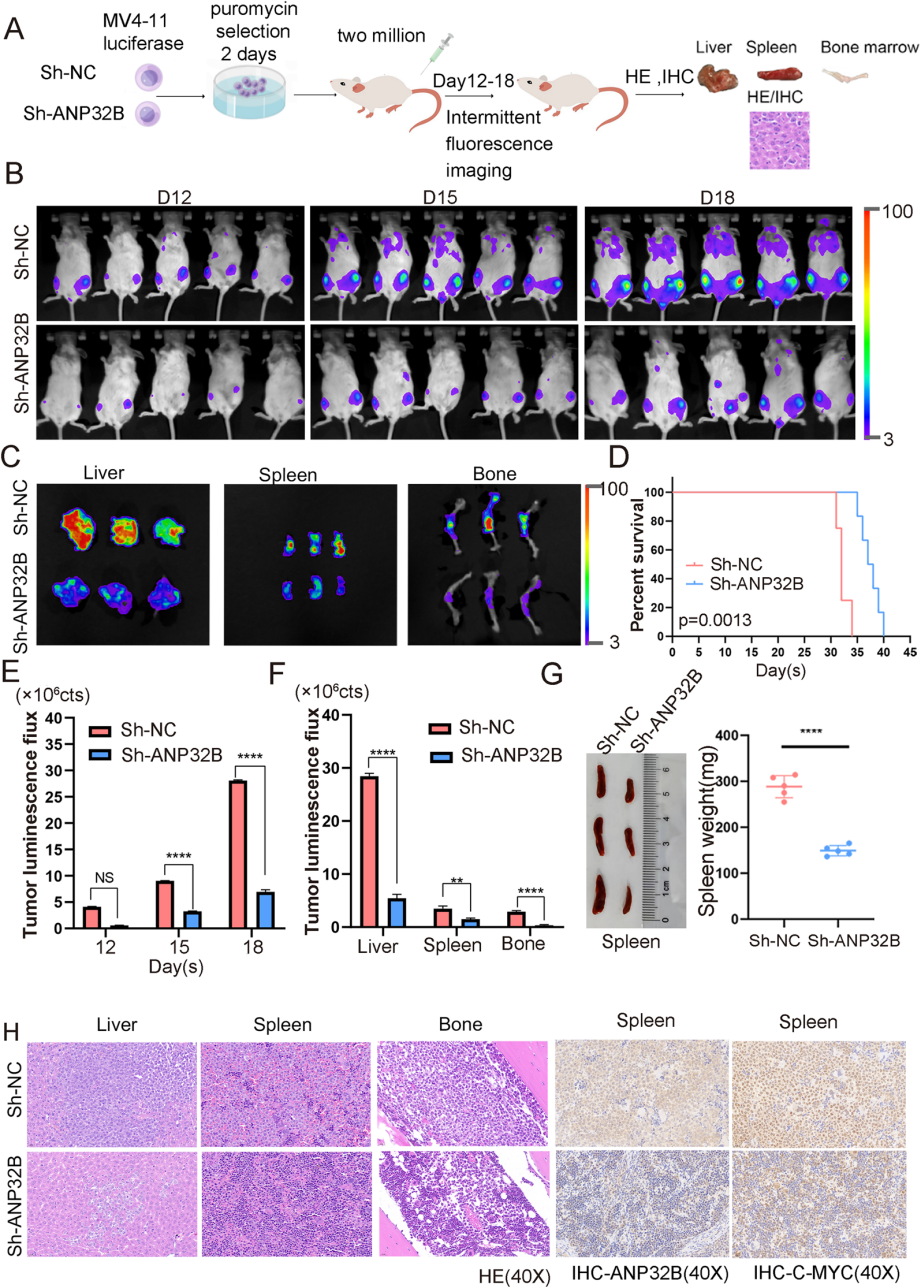
ANP32B knockdown suppresses AML cells growth in vivo. **A** Schematic diagram illustrating the in vivo experimental setup. **B** Relevant bioluminescence imaging of the ANP32B knockdown group and control group on D12, D15, and D18 days. **C** Bioluminescent signal of organs at the endpoint in each group. **D** Mice in the ANP32B knockdown group exhibited prolonged survival time compared to the control group. **E** Histogram shows the bioluminescence signal values for both groups of mice at different time points. **F** Histogram shows the bioluminescence signal values of liver, spleen and bone. **G** Comparison of different weight of spleen between the two groups of mice. **H** H&E staining of organs from both groups of mice. **I** Representative IHC staining of ANP32B and C-MYC in spleen

**Fig. 5:**
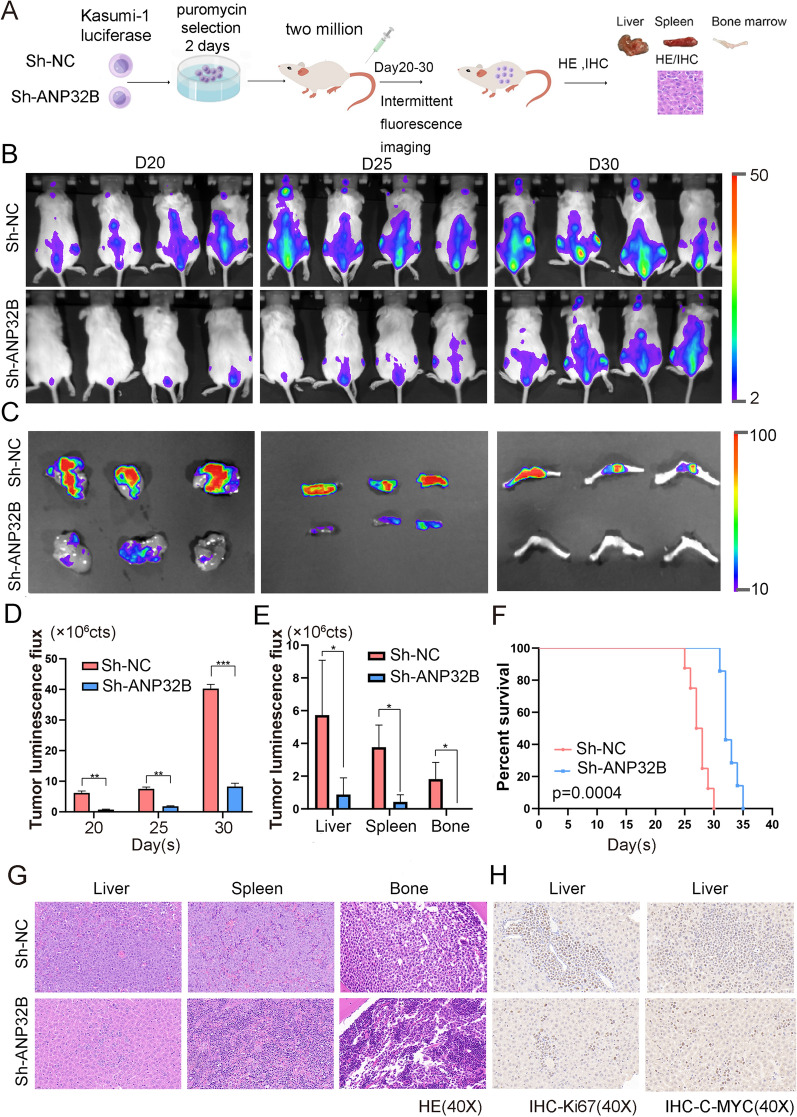
The growth of AML cells in vivo is suppressed upon knockdown of ANP32B. **A** Schematic of the in vivo experiment. **B** Relevant bioluminescence imaging of D20, D25 and D30 days in ANP32B knockdown group and control group. **C** Bioluminescent signal of liver, spleen and bone at the endpoint for each group. **D** Histogram displays the bioluminescence imaging values obtained from both groups of mice at various time intervals. **E** Histogram shows the bioluminescence imaging values of liver, spleen and bone. **F** Survival curves comparing both groups of mice. **G** H&E staining of organs from both groups of mice to assess their histological characteristics. **H** Representative IHC staining of Ki67 and C-MYC in liver tissue

To further investigate the impact of ANP32B on the viability of AML cells in mice, we initially labeled MV4-11 cells with luciferase and subsequently administered ANP32B-targeted shRNA and sh-NC AML cells into NSG mice via the tail vein (Fig. 4A). As depicted in Fig. 4B, the fluorescence signal intensity of mice in the ANP32B knockdown group exhibited a significant decrease. Furthermore, the fluorescence imaging outcomes of liver, spleen, and bone samples of mice indicated a substantial reduction in tumor burden within the ANP32B knockdown group when compared to the control group (Fig. 4C). And the tumor luminous flux histogram indicated a significant decrease in the ANP32B knockdown group compared to the control group (Fig. 4E, and F). Moreover, the comparison of survival time between the two groups of mice revealed that the knockdown of ANP32B gene resulted in an extended lifespan (Fig. 4D). Additionally, the spleen weight was found to be larger in mice from the sh-NC group than ANP32B knockdown group (Fig. 4G). The total weight did not show any statistically significant difference between the two groups. (Additional file 1: Fig. S3A). Furthermore, the H&E staining analysis demonstrated a notable reduction in tumor cells within liver, spleen, and bone marrow samples from the ANP32B knockdown group in comparison to controls (Fig. 4H, Additional file 1: Fig. S3B). Immunohistochemical staining of ANP32B and C-MYC in spleen and liver confirmed that ANP32B knockdown had an inhibitory effect on AML development (Fig. 4I, Additional file 1: Fig. S3C). Subsequent experiments utilizing kasumi-1 cells yielded similar findings (Fig. 5, Additional file 1: Fig. S4), thereby confirming the inhibitory effect of ANP32B knockdown on the viability of AML cells in vivo.

### Knockdown of ANP32B downregulates MYC expression in AML cell lines

**Fig. 6:**
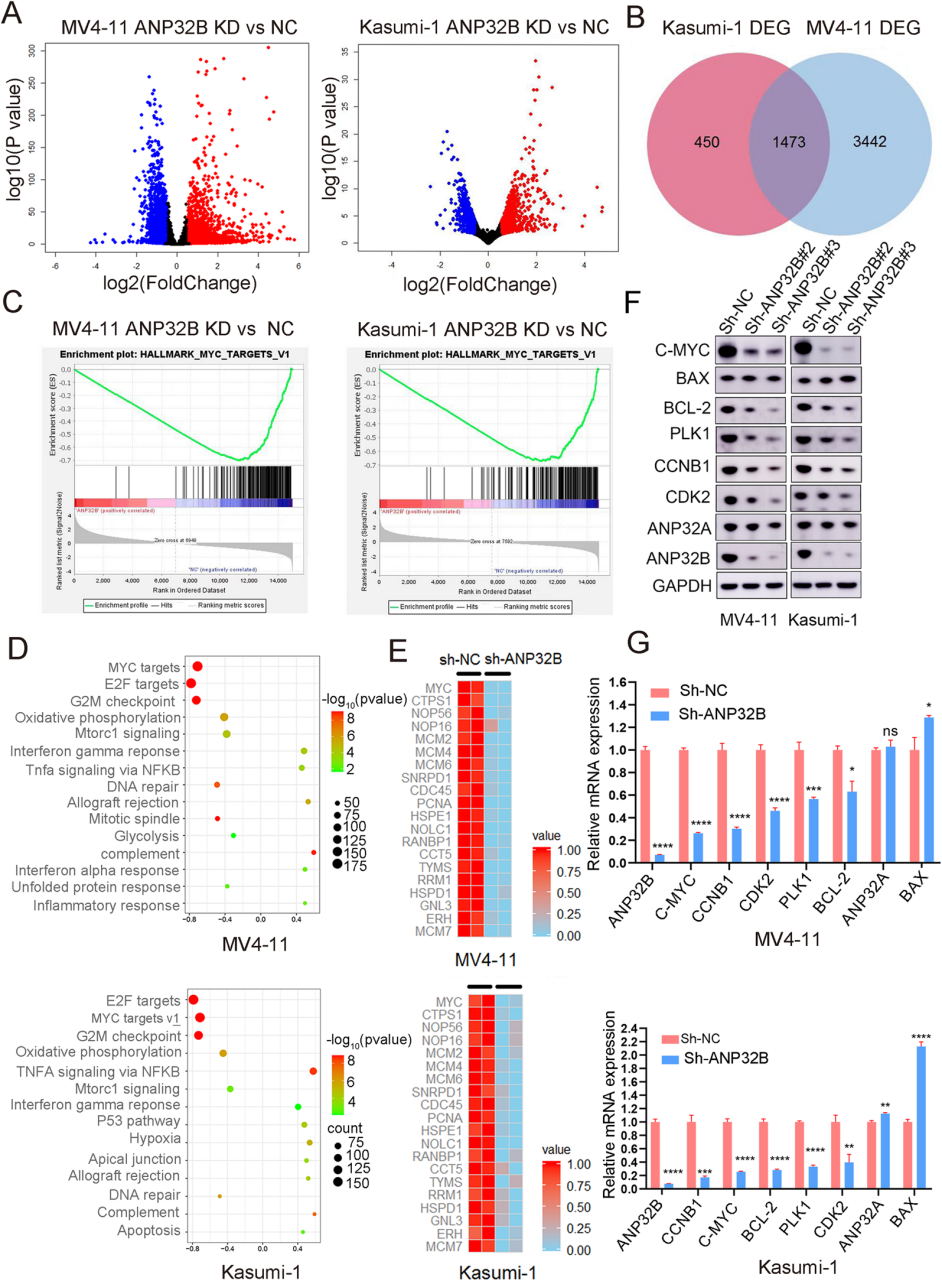
ANP32B effects MYC expression in AML cell lines. **A** Volcano plot analysis revealed differentially expressed genes obtained from RNA-seq data between the ANP32B knockdown and the control groups. **B** After ANP32B knockdown, MV4-11 and Kasumi-1 cells exhibited enrichment of down-regulated genes, resulting in 1473 common genes being identified, including MYC. **C** GSEA plots demonstrated gene enrichment in HALLMARK_MYC signaling pathways in AML cells treated with ANP32B knockdown. **D** KEGG pathway enrichment analysis. **E** Heatmap view displayed the top downregulated genes in MV4-11 and Kasumi-1 cells treated with ANP32B knockdown. **F** Western blotting analysis showed that the C-MYC, ANP32B, CCNB1, CDK2, BCL-2 and PLK1 were downregulated in MV4-11 and Kasumi-1 cells after ANP32B knockdown. **G** ANP32B knockdown led to downregulate of MYC, CCNB1, CDK2, BCL-2 and PLK1 in MV4-11 and Kasumi-1 cells, determined using qRT-PCR

The potential mechanism of ANP32B was investigated by employing RNA-seq to conduct a comprehensive screening and analysis of the target genes associated with ANP32B. In comparison to the control group, a total of 4915 genes exhibited down-regulation in MV4-11 cells after ANP32B was knocked down (Additional file 1: Table S3), while 1923 genes displayed significant down-regulation in kasumi-1 cells (Fig. 6A, Additional file 1: Table S4). A comparative examination unveiled 1473 gene overlaps between the two cell lines, including MYC (Fig. 6B). Subsequent GSEA and KEGG pathway enrichment analyses demonstrated that, following ANP32B knockdown in both MV4-11 and Kasumi-1 cells, the genes were predominantly enriched in the MYC, G2M, and E2F signaling pathways (Fig. 6C and D). The heatmap depicted the primary down-regulated genes within the MYC signaling pathway after ANP32B knockdown (Fig. 6E). The findings imply that MYC potentially participates in the functional regulation of ANP32B as a downstream target gene.

In order to validate the downregulation of genes in the MYC and G2M signaling pathways, we conducted an analysis of the expression of several downstream genes using WB and qRT-PCR. As anticipated, the results from both WB and qRT-PCR confirmed that the levels of MYC, CCNB1, BCL-2, PLK1, and CDK2 proteins and RNA in MV4-11 and Kasumi-1 cells decreased upon knockdown of ANP32B. Notably, the expression of ANP32A remained unchanged (Fig. 6F, G). These findings suggest that the interference of ANP32B knockdown leads to a reduction in C-MYC protein levels in AML cells, thereby impacting the MYC pathway, which aligns with the RNA sequencing results.

### Knockdown of ANP32B decrease the levels of lysine 27 on histone 3 (H3K27ac) in AML cell lines

**Fig. 7:**
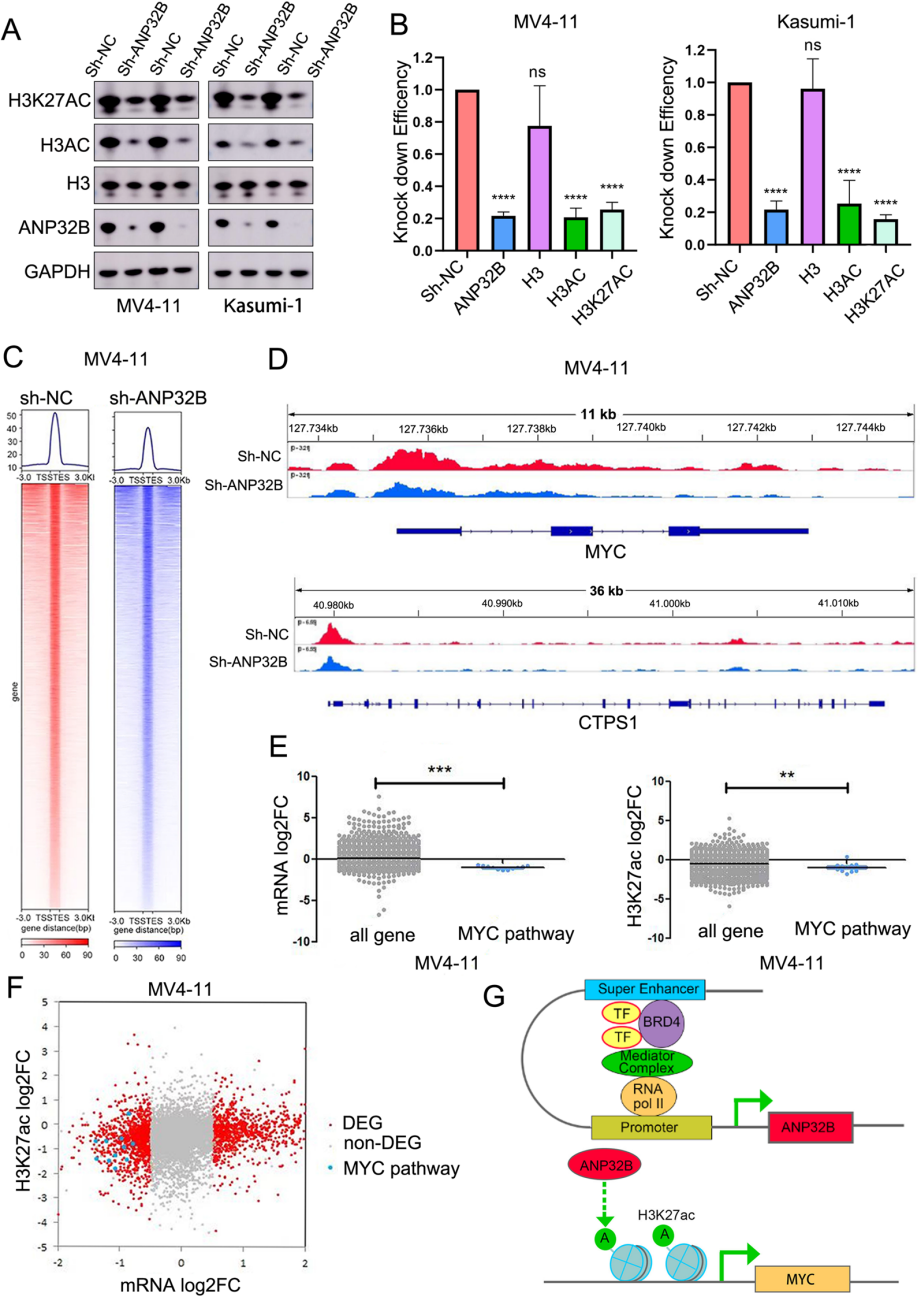
The Knockdown of ANP32B Resulted in a Significant Decrease in the Levels of Acetylated Histone H3 in AML Cell Lines. **A**, **B** Western blotting analysis showed that ANP32B knockdown caused a substantial decrease in global acetyl-H3 and acetylation of histone H3k27. **C** Heat map generated by CUT&Tag data analysis. CUT&Tag technology showed that a decrease in the overall occupancy of H3K27ac across the entire genome following ANP32B knockdown. **D** IGV visual analysis showed a reduction in H3K27ac signaling in genes involved in the MYC signaling pathway. **E** Box plots for log2 fold-change in mRNA expression of genes and H3K27ac enrichment signals with all genes enrichment (grey) or MYC pathway enrichment (blue). **F** The volcano plot analysis showed that the MYC pathway co-existed in log2 fold-change in mRNA expression and H3K27ac enrichment signals. **G** Schematic representation of ANP32B in the pathogenesis of AML

It has been observed that ANP32A possesses the ability to modulate gene expression via epigenetic mechanisms, specifically by decreasing the levels of histone H3 acetylation [46]. However, the potential for ANP32B to operate through a similar mechanism remains undetermined. Consequently, we conducted further investigations to elucidate the manner in which ANP32B regulates C-MYC expression in acute myeloid leukemia cells. Western blot analysis demonstrated that the knockdown of ANP32B in MV4-11 and Kasumi-1 cell lines had minimal impact on H3 protein expression, but resulted in a reduction in the acetylation level of H3, especially the level of lysine 27 on histone 3 (H3K27ac) (Fig. 7A and B).

To further understand the regulatory association between H3K27ac and C-MYC expression subsequent to ANP32B knockdown, we employed CUT&TAG technology to examine the impact of H3K27ac downregulation on C-MYC gene transcription. The findings revealed a decrease in the overall occupancy of H3K27ac across the entire genome following ANP32B knockdown (Fig. 7C, Additional file 1: Tables S5, S6). IGV visual analysis demonstrated a significant reduction in H3K27ac signals for key genes involved in the MYC signaling pathway, namely MYC, CTPS1, MCM6, RANBP1, TRMT2A, and SNORA77B, subsequent to ANP32B knockdown (Fig. 7D and Additional file 1: Fig. S5). Simultaneously, a substantial decrease in the expression of these genes was observed subsequent to the knockdown of ANP32B. These findings imply that the reduction in H3K27ac levels is likely to exert an influence on gene transcription, particularly the MYC pathway (Fig. 7E and F). Consequently, it can be inferred that ANP32B, acting as a super enhancer, potentially regulate the expression of MYC via epigenetic regulation of histone acetylation, thereby impacting the occurrence and progression of AML (Fig. 7G).

## Discussion

AML is a heterogeneous disease characterized by diverse oncogenic drivers [24]. In AML, it has been proved that super enhancers facilitate the upregulation of oncogenes and initiate the development of leukemia. In the current investigation, we performed H3K27ac ChIP-seq analysis in AML samples and cell lines, and screened out ANP32B as a gene regulated by super enhancers in AML. We utilized a publicly available transcriptomic dataset (GSE114868) [23] to compare the expression profiles of AML cases with those of healthy controls. Our findings revealed a significant upregulation of ANP32B in AML. Moreover, patients exhibiting elevated levels of ANP32B expression experienced unfavorable clinical outcomes. Notably, our study demonstrated that the knockdown of ANP32B impeded cell proliferation both in vitro and in vivo. Additionally, ANP32B knockdown markedly augmented apoptosis and induced cell cycle arrest in AML cells. These outcomes strongly suggest the pivotal role of ANP32B in AML.

In this study, we have made a novel discovery that the knockdown of ANP32B leads to the inhibition of C-MYC expression, while overexpression of ANP32B increased C-MYC expression (Additional file 1: Fig. S6), indicating that ANP32B may serve as an upstream regulator of the C-MYC gene. MYC, a well-studied oncogene, primarily functions as a transcriptional regulator. The MYC protein governs a diverse array of cellular processes, encompassing cell growth, cell cycle regulation, cellular differentiation, programmed cell death, blood vessel formation, metabolic pathways, DNA repair mechanisms, protein synthesis, immune responses, and stem cell formation [25–27]. MYC overexpression has been observed in the majority of acute myeloid leukemia (AML) patients [28]. Extensive research has demonstrated the association between MYC and the proliferation and differentiation of AML cells [29]. Studies have indicated that the suppression of MYC expression can effectively reverse tumor progression [30].

Recently, various drug-based approaches have been proposed to target MYC and impede tumor growth [31–33]. The primary focus of these investigations has been on inhibiting MYC-MAX interactions, suppressing MYC expression, and targeting synthetic lethal genes that exhibit MYC overexpression. BRD4 has recently been discovered to bind to the MYC promoter and exert regulatory control over its transcription [35]. Numerous BET inhibitors, particularly the BRD4 inhibitor JQ1, have demonstrated the ability to downregulate MYC and impede tumor growth in various animal models with MYC activation [36–38]. A study has demonstrated that APTO-253 can stabilize the G4 structure within the MYC promoter, resulting in reduced MYC expression, cell cycle arrest, and induction of apoptosis in AML cell lines [39]. Furthermore, studies have highlighted the significance of HDAC inhibitors in modulating MYC regulation and acetylation, thereby presenting promising therapeutic targets [34].

Histone lysine acetyltransferases (HATs) and histone deacetylases (HDACs) control histone acetylation. Histone methylation and acetylation are two essential mechanisms involved in the epigenetic regulation of gene expression [40]. The process of histone acetylation plays a critical role in gene transcription, chromatin structure, and DNA repair [41]. Specifically, lysine acetylation promotes open chromatin conformations and gene activation [42]. HDAC inhibitors (HDACi) have been shown to activate tumor suppressor genes and enhance the elimination of tumor cells [43]. These inhibitors have been assessed in clinical trials for adult AML patients, but their effectiveness has been limited when used alone or in conjunction with chemotherapy [44]. While there have been no direct reports on the regulation of histone modification by ANP32B, its counterpart ANP32A, another member of the ANP32 protein family, has been implicated in the regulation of histone acetylation in leukemia pathogenesis. It has been found to associate with the acetyltransferase inhibitor complex (INHAT), which includes the Set/TAF-Ibeta cancer protein [45]. Within this complex, ANP32A effectively inhibits the HAT activity of p300/CBP and PCAF through histone shielding. Given the structural similarity between ANP32B and ANP32A, it is reasonable to speculate that ANP32B may also be involved in histone modification and functional regulation as part of a protein complex. However, further studies are needed to elucidate the specific mechanisms by which ANP32B participates in histone modification and its functional consequences.

The presence of leukemia-associated fusion proteins has been observed to impede gene expression through the recruitment of HDACs. However, this hindrance can be alleviated by inhibiting HDACs, thereby inducing differentiation of leukemic blasts [46]. ANP32A, a member of the ANP32 family, has been identified as a contributor to leukemia development by modulating acetyl-H3 levels on crucial pathways such as lipid metabolism [9]. We formulated a hypothesis regarding the potential impact of ANP32B on histone acetylation levels. Our findings from western blot analysis revealed that the knockout of ANP32B in MV4-11 and Kasumi-1 cell lines had minimal influence on the overall expression of total H3 protein. However, there was a notable decrease in the acetylation level of H3, particularly H3K27ac. The results obtained from CUT&TAG analysis demonstrated that ANP32B played a role in modulating the enrichment of histone H3K27ac within the promoter region of MYC, as illustrated in Fig. 7. CUT&TAG analysis revealed a decrease in the overall occupancy of H3K27ac across the entire genome following ANP32B knockdown. When comparing CUT&TAG technology to western blot exposure, the latter is more sensitive and may amplify differences, which likely explains the inconsistency observed between the two assays. These findings confirm the ability of ANP32B to regulate gene expression through epigenetic mechanisms. Hu et al. also reported that the NAT10-ac4C-ANP32B axis can regulate the chromatin landscape of downstream genes, such as key regulators of the Wnt and TGF β pathways, and plays a crucial role in determining the fate of stem cells. Furthermore, ANP32B is involved in the dynamic regulation of chromatin, including histone bivalent modifications (H3K4me3, H3K27me3), and chromatin accessibility [47]. These findings suggest that ANP32B exerts epigenetic regulation on MYC expression. However, we have only explained part of the mechanism of ANP32B in epigenetic regulation of AML occurrence and development, and the exact mechanism of ANP32B in chromosome modification remains to be further studied.

Recent research has elucidated the substantial involvement of epigenetic dysregulation in the development of acute myeloid leukemia (AML), particularly in pediatric cases where recurrent somatic genetic alterations may impede proper epigenetic regulation [7]. Given the reversible nature of epigenetic modifications, there is considerable potential for targeted therapy utilizing specific inhibitors [8]. Consequently, the field of epigenetic therapy has emerged as a promising therapeutic approach, with numerous novel inhibitors currently being employed in the treatment of adult AML [48]. Thus, it is imperative to acknowledge the crucial role of epigenetic alterations and the potential therapeutic interventions they offer in the context of pediatric AML. This will facilitate the development of precision therapy and enhance outcomes of children with AML.

## Conclusion

Our study has provided evidence to support the notion that super-enhancer related gene ANP32B plays a significant role in promoting tumor proliferation in acute myeloid leukemia (AML). The downregulation of ANP32B has been observed to induce cell cycle arrest and promote apoptosis in AML cell lines. Furthermore, mechanistic analysis suggests that ANP32B may epigenetically regulate the expression of MYC through histone H3K27 acetylation. These findings highlight the potential of ANP32B as a promising biomarker for prognostic evaluation and a potential therapeutic target for patients with acute myeloid leukemia (AML).

### Supplementary Information

**Additional file 1. Figure S1.** Western blotting analysis showed that ANP32B protein levels were downregulated in MV4-11 and Kasumi-1 cells after BRD4 knockdown. **Figure S2.** The knockdown level of ANP32B in MV4-11 and Kasumi-1 cells was verified by qPCR. **Figure S3.** The knockdown of ANP32B inhibited the growth of MV4-11 cells. A. Monitoring of body weight of the two groups of mice. B. Representative images of H&E staining analysis of liver in two groups of mice. C. Representative images of IHC staining of mice liver. **Figure S4.** The knockdown of ANP32B inhibited the growth of Kasumi-1 cells. A. Different size and weight of spleen, from sh-NC or sh-ANP32B mices. B. Representative images of IHC staining of mice spleen. **Figure S5.** IGV visual analysis showed a reduction in H3K27ac signaling in genes involved in the MYC signaling pathway. **Figure S6.** ANP32B is positively correlated with C-MYC expression. A. Western blotting analysis showed that ANP32B overexpression was established successfully. B. Western blotting analysis showed that ANP32B was positively correlated with C-MYC expression. **Table S1.** The primer sequences used in this study. **Table S2.** Super-enhancers identified in each of the 11 AML samples. **Table S3.** Deferentially expressed genes identified by RNA-Seq of MV4-11 cell after ANP32B knockdown. **Table S4.** Deferentially expressed genes identified by RNA-Seq of Kasumi-1 cell after ANP32B knockdown. **Table S5.** In the control group, peaks called by ChIP-Seq of H3K27ac in MV4-11 cell. **Table S6.** In the ANP32B knockdown group, peaks called by ChIP-Seq of H3K27ac in MV4-11 cell.

## Data Availability

The data that support the findings of this study are available on reasonable request from the corresponding author. RNA‑seq and CUT-TAG data have been submitted to the GEO database with Accession Number GSE242850.
