# Supplementary figures and images for: Super enhancer related gene ANP32B promotes the proliferation of acute myeloid leukemia by enhancing MYC through histone acetylation
Source: Cancer Cell Int. 2024 Feb 22;24:81. doi: 10.1186/s12935-024-03271-y (PMC10882810; doi:10.1186/s12935-024-03271-y)

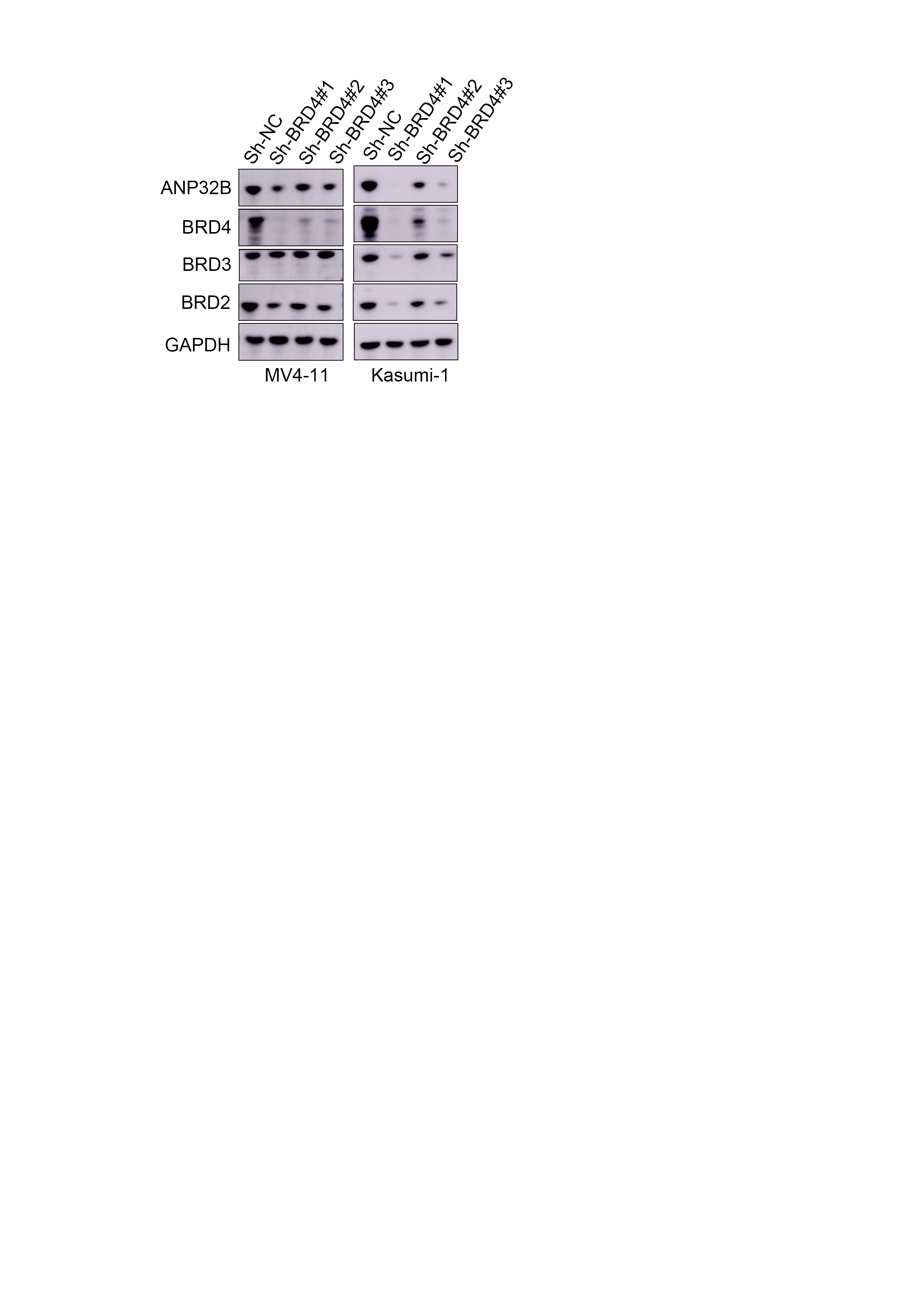

Supplement: Supplementary file 1 — Additional file 1. Figure S1. Western blotting analysis showed that ANP32B protein levels were downregulated in MV4-11 and Kasumi-1 cells after BRD4 knockdown. Figure S2. The knockdown level of ANP32B in MV4-11 and Kasumi-1 cells was verified by qPCR. Figure S3. The knockdown of ANP32B inhibited the growth of MV4-11 cells. A. Monitoring of body weight of the two groups of mice. B. Representative images of H&E staining analysis of liver in two groups of mice. C. Representative images of IHC staining of mice liver. Figure S4. The knockdown of ANP32B inhibited the growth of Kasumi-1 cells. A. Different size and weight of spleen, from sh-NC or sh-ANP32B mices. B. Representative images of IHC staining of mice spleen. Figure S5. IGV visual analysis showed a reduction in H3K27ac signaling in genes involved in the MYC signaling pathway. Figure S6. ANP32B is positively correlated with C-MYC expression. A. Western blotting analysis showed that ANP32B overexpression was established successfully. B. Western blotting analysis showed that ANP32B was positively correlated with C-MYC expression. Table S1. The primer sequences used in this study. Table S2. Super-enhancers identified in each of the 11 AML samples. Table S3. Deferentially expressed genes identified by RNA-Seq of MV4-11 cell after ANP32B knockdown. Table S4. Deferentially expressed genes identified by RNA-Seq of Kasumi-1 cell after ANP32B knockdown. Table S5. In the control group, peaks called by ChIP-Seq of H3K27ac in MV4-11 cell. Table S6. In the ANP32B knockdown group, peaks called by ChIP-Seq of H3K27ac in MV4-11 cell. [file 12935_2024_3271_MOESM1_ESM.zip › New folder/Supplementary Figure 1.png]

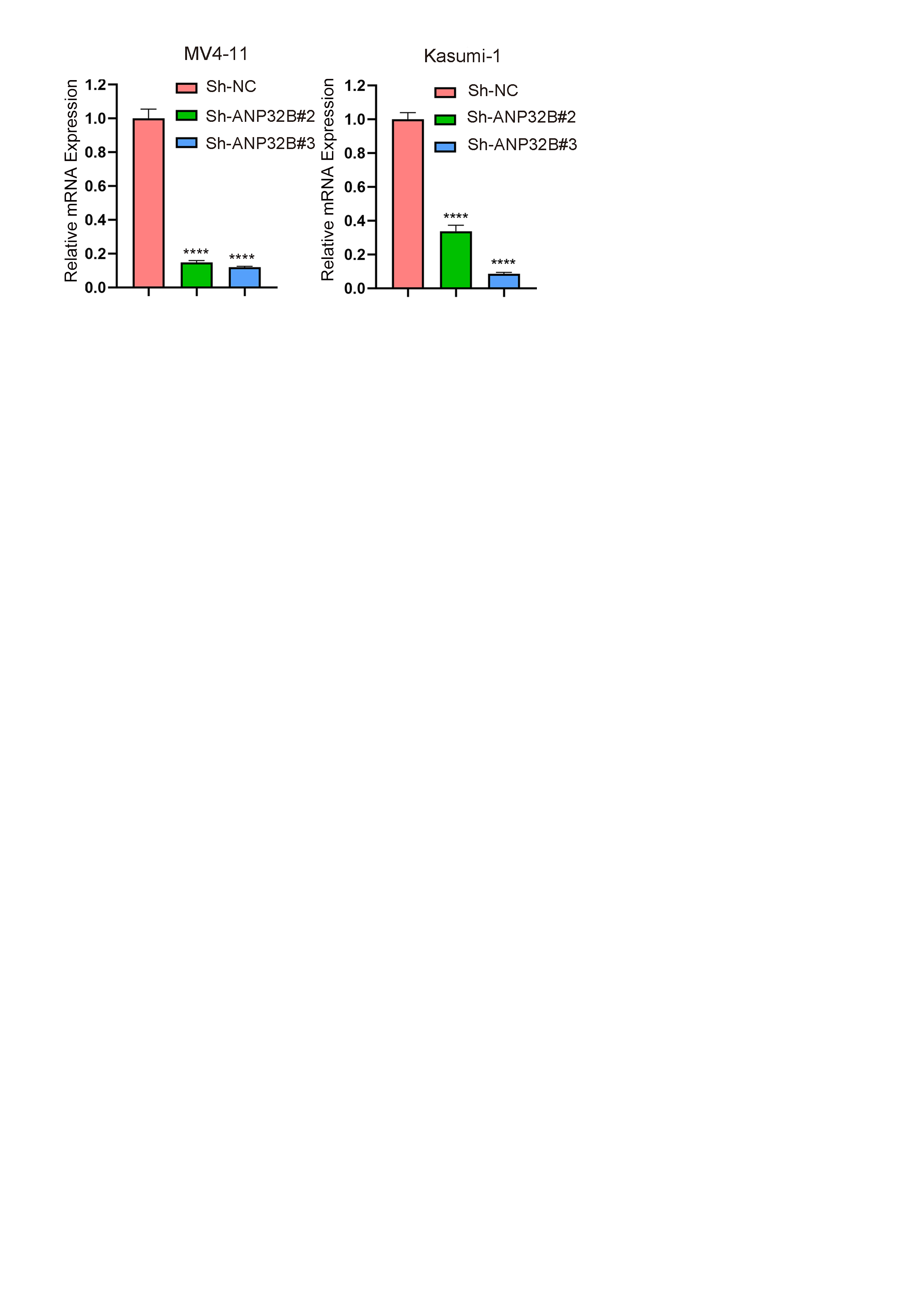

Supplement: Supplementary file 1 — Additional file 1. Figure S1. Western blotting analysis showed that ANP32B protein levels were downregulated in MV4-11 and Kasumi-1 cells after BRD4 knockdown. Figure S2. The knockdown level of ANP32B in MV4-11 and Kasumi-1 cells was verified by qPCR. Figure S3. The knockdown of ANP32B inhibited the growth of MV4-11 cells. A. Monitoring of body weight of the two groups of mice. B. Representative images of H&E staining analysis of liver in two groups of mice. C. Representative images of IHC staining of mice liver. Figure S4. The knockdown of ANP32B inhibited the growth of Kasumi-1 cells. A. Different size and weight of spleen, from sh-NC or sh-ANP32B mices. B. Representative images of IHC staining of mice spleen. Figure S5. IGV visual analysis showed a reduction in H3K27ac signaling in genes involved in the MYC signaling pathway. Figure S6. ANP32B is positively correlated with C-MYC expression. A. Western blotting analysis showed that ANP32B overexpression was established successfully. B. Western blotting analysis showed that ANP32B was positively correlated with C-MYC expression. Table S1. The primer sequences used in this study. Table S2. Super-enhancers identified in each of the 11 AML samples. Table S3. Deferentially expressed genes identified by RNA-Seq of MV4-11 cell after ANP32B knockdown. Table S4. Deferentially expressed genes identified by RNA-Seq of Kasumi-1 cell after ANP32B knockdown. Table S5. In the control group, peaks called by ChIP-Seq of H3K27ac in MV4-11 cell. Table S6. In the ANP32B knockdown group, peaks called by ChIP-Seq of H3K27ac in MV4-11 cell. [file 12935_2024_3271_MOESM1_ESM.zip › New folder/Supplementary Figure 2.png]

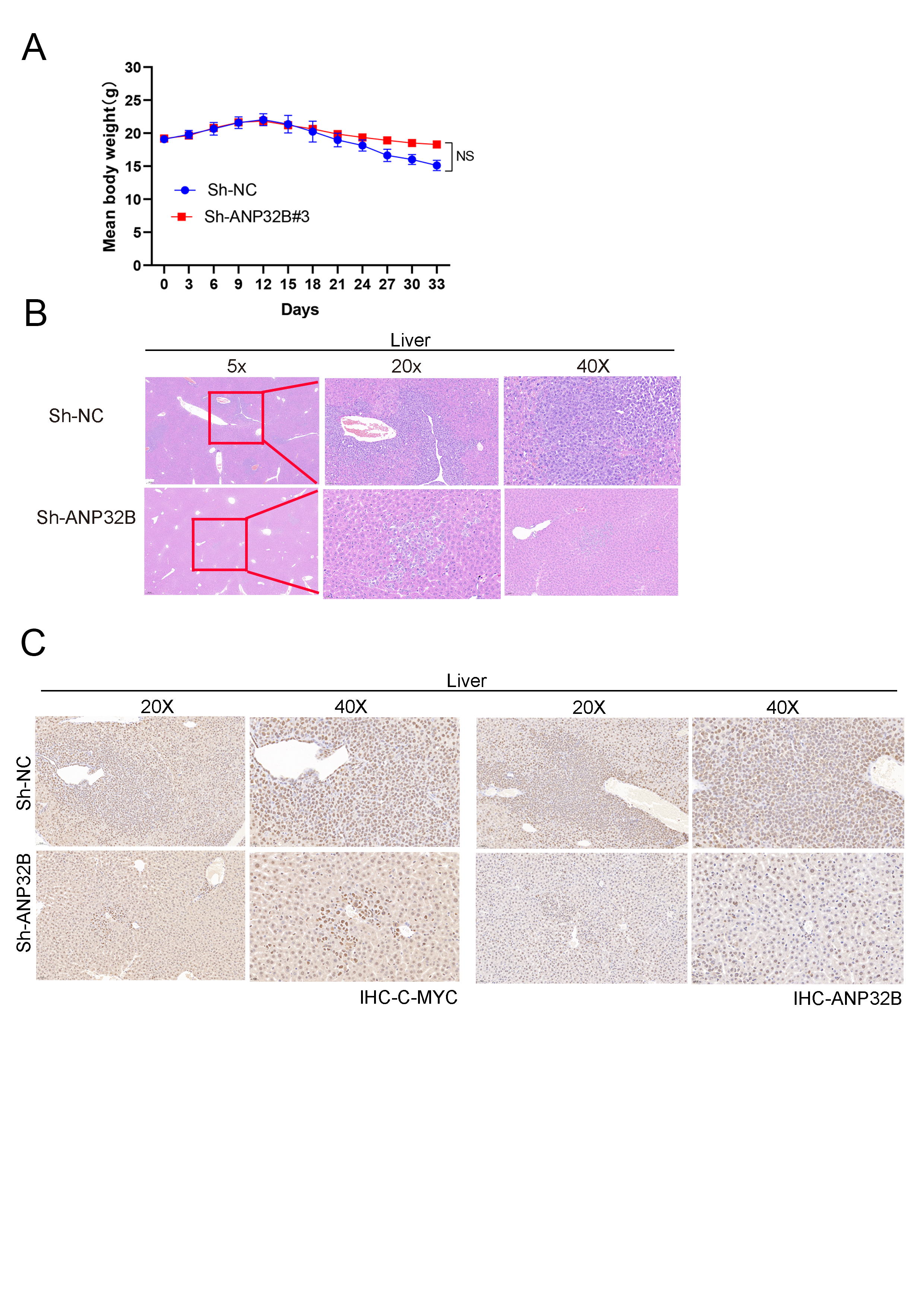

Supplement: Supplementary file 1 — Additional file 1. Figure S1. Western blotting analysis showed that ANP32B protein levels were downregulated in MV4-11 and Kasumi-1 cells after BRD4 knockdown. Figure S2. The knockdown level of ANP32B in MV4-11 and Kasumi-1 cells was verified by qPCR. Figure S3. The knockdown of ANP32B inhibited the growth of MV4-11 cells. A. Monitoring of body weight of the two groups of mice. B. Representative images of H&E staining analysis of liver in two groups of mice. C. Representative images of IHC staining of mice liver. Figure S4. The knockdown of ANP32B inhibited the growth of Kasumi-1 cells. A. Different size and weight of spleen, from sh-NC or sh-ANP32B mices. B. Representative images of IHC staining of mice spleen. Figure S5. IGV visual analysis showed a reduction in H3K27ac signaling in genes involved in the MYC signaling pathway. Figure S6. ANP32B is positively correlated with C-MYC expression. A. Western blotting analysis showed that ANP32B overexpression was established successfully. B. Western blotting analysis showed that ANP32B was positively correlated with C-MYC expression. Table S1. The primer sequences used in this study. Table S2. Super-enhancers identified in each of the 11 AML samples. Table S3. Deferentially expressed genes identified by RNA-Seq of MV4-11 cell after ANP32B knockdown. Table S4. Deferentially expressed genes identified by RNA-Seq of Kasumi-1 cell after ANP32B knockdown. Table S5. In the control group, peaks called by ChIP-Seq of H3K27ac in MV4-11 cell. Table S6. In the ANP32B knockdown group, peaks called by ChIP-Seq of H3K27ac in MV4-11 cell. [file 12935_2024_3271_MOESM1_ESM.zip › New folder/Supplementary Figure 3.png]

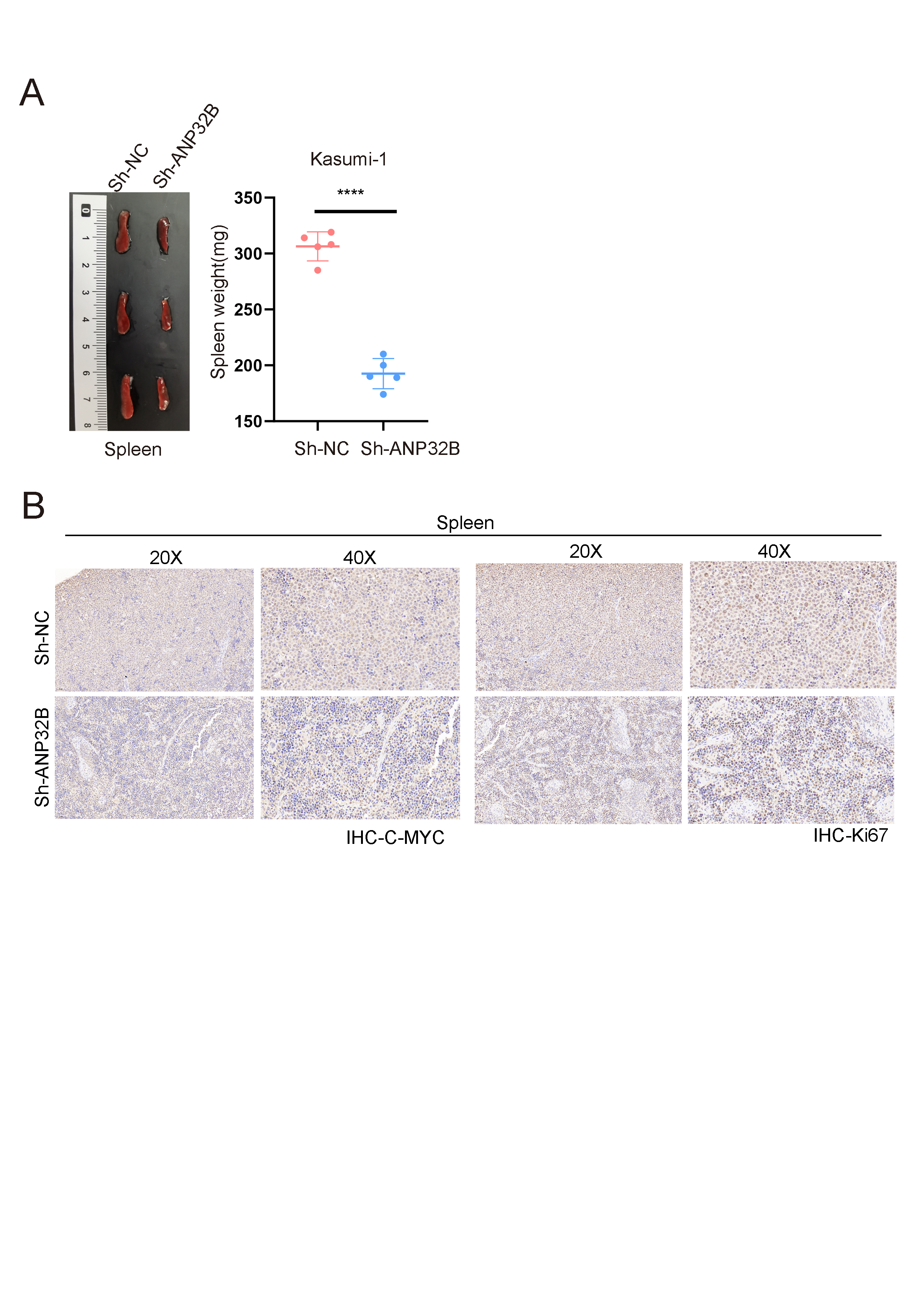

Supplement: Supplementary file 1 — Additional file 1. Figure S1. Western blotting analysis showed that ANP32B protein levels were downregulated in MV4-11 and Kasumi-1 cells after BRD4 knockdown. Figure S2. The knockdown level of ANP32B in MV4-11 and Kasumi-1 cells was verified by qPCR. Figure S3. The knockdown of ANP32B inhibited the growth of MV4-11 cells. A. Monitoring of body weight of the two groups of mice. B. Representative images of H&E staining analysis of liver in two groups of mice. C. Representative images of IHC staining of mice liver. Figure S4. The knockdown of ANP32B inhibited the growth of Kasumi-1 cells. A. Different size and weight of spleen, from sh-NC or sh-ANP32B mices. B. Representative images of IHC staining of mice spleen. Figure S5. IGV visual analysis showed a reduction in H3K27ac signaling in genes involved in the MYC signaling pathway. Figure S6. ANP32B is positively correlated with C-MYC expression. A. Western blotting analysis showed that ANP32B overexpression was established successfully. B. Western blotting analysis showed that ANP32B was positively correlated with C-MYC expression. Table S1. The primer sequences used in this study. Table S2. Super-enhancers identified in each of the 11 AML samples. Table S3. Deferentially expressed genes identified by RNA-Seq of MV4-11 cell after ANP32B knockdown. Table S4. Deferentially expressed genes identified by RNA-Seq of Kasumi-1 cell after ANP32B knockdown. Table S5. In the control group, peaks called by ChIP-Seq of H3K27ac in MV4-11 cell. Table S6. In the ANP32B knockdown group, peaks called by ChIP-Seq of H3K27ac in MV4-11 cell. [file 12935_2024_3271_MOESM1_ESM.zip › New folder/Supplementary Figure 4.png]

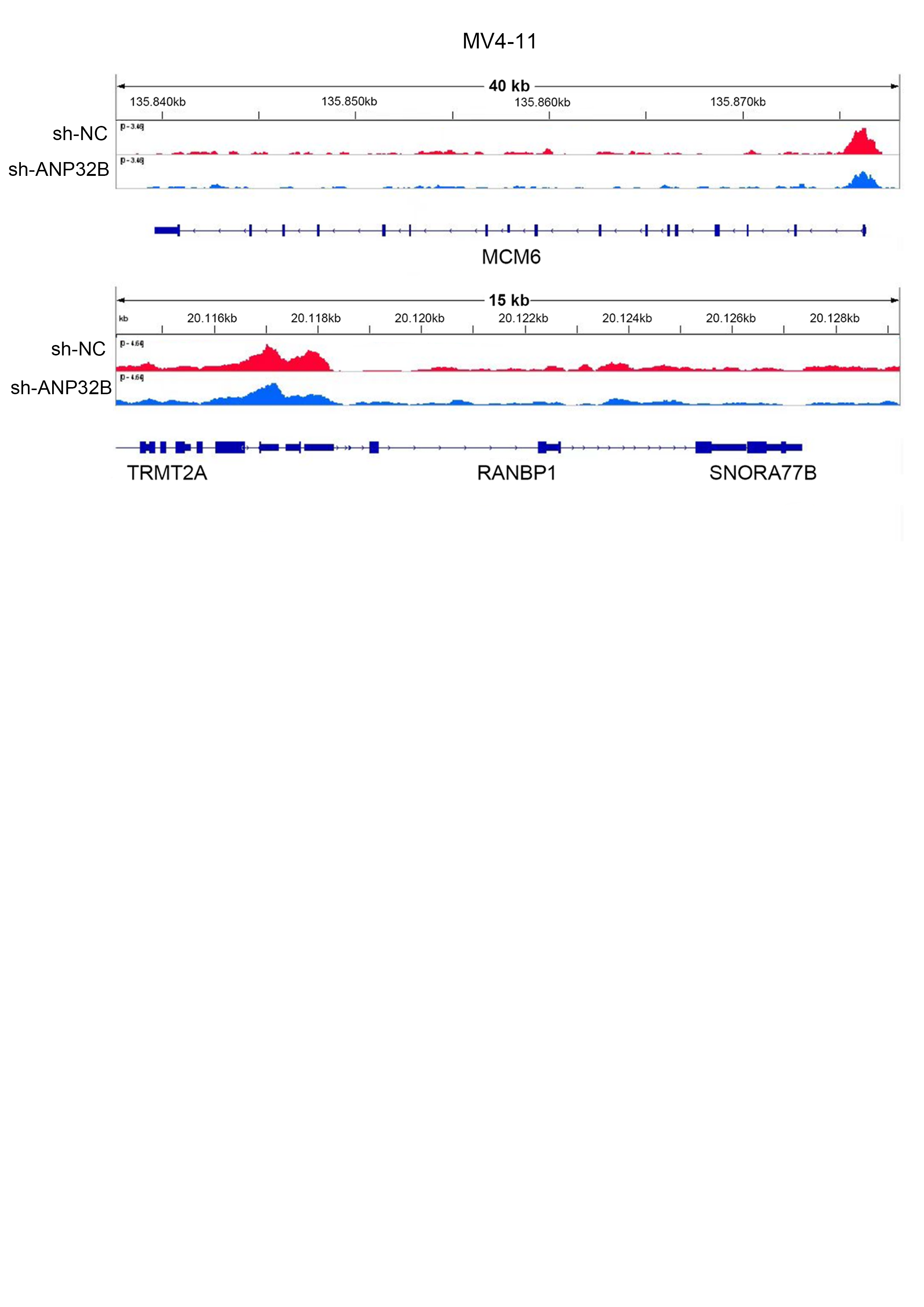

Supplement: Supplementary file 1 — Additional file 1. Figure S1. Western blotting analysis showed that ANP32B protein levels were downregulated in MV4-11 and Kasumi-1 cells after BRD4 knockdown. Figure S2. The knockdown level of ANP32B in MV4-11 and Kasumi-1 cells was verified by qPCR. Figure S3. The knockdown of ANP32B inhibited the growth of MV4-11 cells. A. Monitoring of body weight of the two groups of mice. B. Representative images of H&E staining analysis of liver in two groups of mice. C. Representative images of IHC staining of mice liver. Figure S4. The knockdown of ANP32B inhibited the growth of Kasumi-1 cells. A. Different size and weight of spleen, from sh-NC or sh-ANP32B mices. B. Representative images of IHC staining of mice spleen. Figure S5. IGV visual analysis showed a reduction in H3K27ac signaling in genes involved in the MYC signaling pathway. Figure S6. ANP32B is positively correlated with C-MYC expression. A. Western blotting analysis showed that ANP32B overexpression was established successfully. B. Western blotting analysis showed that ANP32B was positively correlated with C-MYC expression. Table S1. The primer sequences used in this study. Table S2. Super-enhancers identified in each of the 11 AML samples. Table S3. Deferentially expressed genes identified by RNA-Seq of MV4-11 cell after ANP32B knockdown. Table S4. Deferentially expressed genes identified by RNA-Seq of Kasumi-1 cell after ANP32B knockdown. Table S5. In the control group, peaks called by ChIP-Seq of H3K27ac in MV4-11 cell. Table S6. In the ANP32B knockdown group, peaks called by ChIP-Seq of H3K27ac in MV4-11 cell. [file 12935_2024_3271_MOESM1_ESM.zip › New folder/Supplementary Figure 5.png]

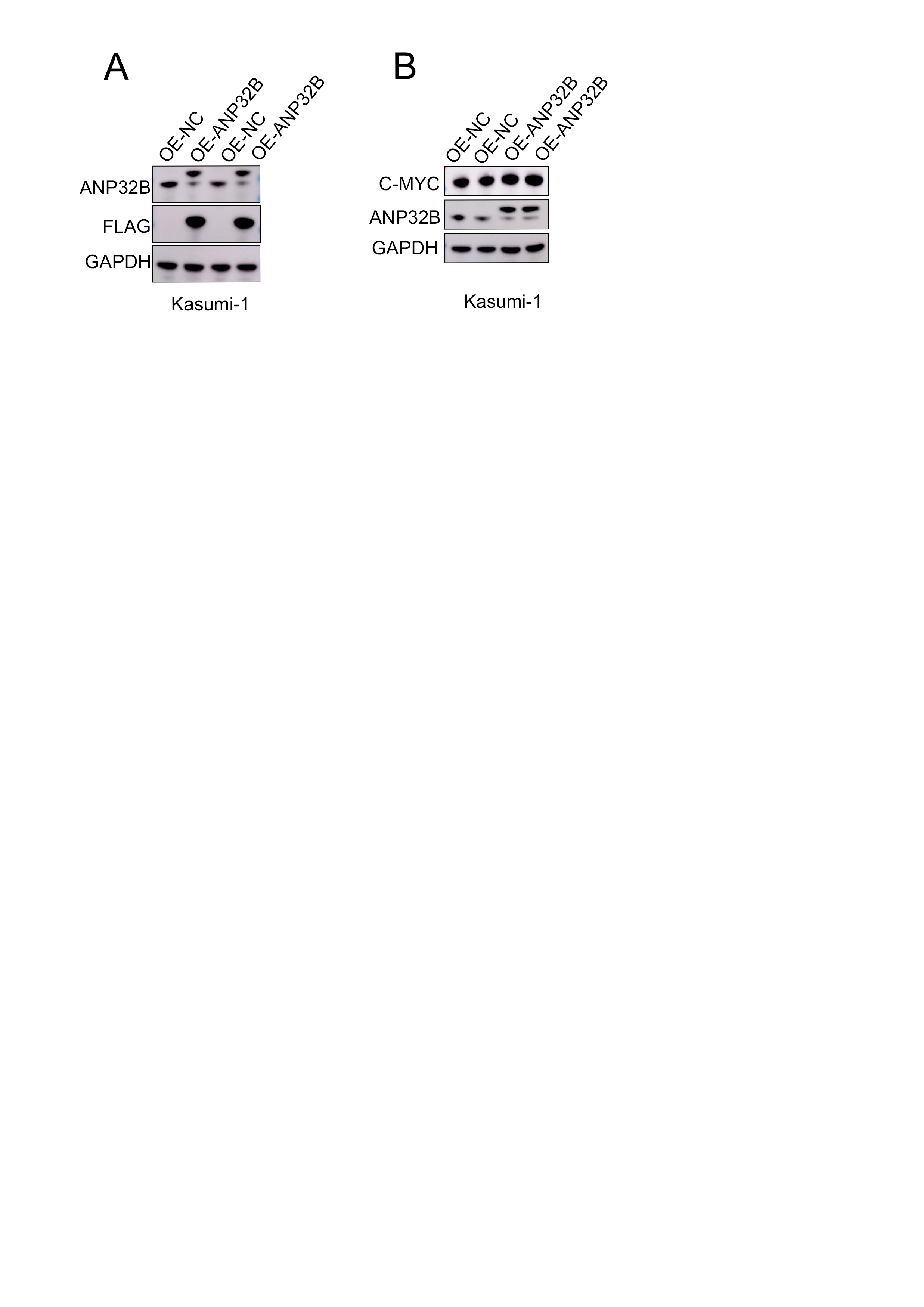

Supplement: Supplementary file 1 — Additional file 1. Figure S1. Western blotting analysis showed that ANP32B protein levels were downregulated in MV4-11 and Kasumi-1 cells after BRD4 knockdown. Figure S2. The knockdown level of ANP32B in MV4-11 and Kasumi-1 cells was verified by qPCR. Figure S3. The knockdown of ANP32B inhibited the growth of MV4-11 cells. A. Monitoring of body weight of the two groups of mice. B. Representative images of H&E staining analysis of liver in two groups of mice. C. Representative images of IHC staining of mice liver. Figure S4. The knockdown of ANP32B inhibited the growth of Kasumi-1 cells. A. Different size and weight of spleen, from sh-NC or sh-ANP32B mices. B. Representative images of IHC staining of mice spleen. Figure S5. IGV visual analysis showed a reduction in H3K27ac signaling in genes involved in the MYC signaling pathway. Figure S6. ANP32B is positively correlated with C-MYC expression. A. Western blotting analysis showed that ANP32B overexpression was established successfully. B. Western blotting analysis showed that ANP32B was positively correlated with C-MYC expression. Table S1. The primer sequences used in this study. Table S2. Super-enhancers identified in each of the 11 AML samples. Table S3. Deferentially expressed genes identified by RNA-Seq of MV4-11 cell after ANP32B knockdown. Table S4. Deferentially expressed genes identified by RNA-Seq of Kasumi-1 cell after ANP32B knockdown. Table S5. In the control group, peaks called by ChIP-Seq of H3K27ac in MV4-11 cell. Table S6. In the ANP32B knockdown group, peaks called by ChIP-Seq of H3K27ac in MV4-11 cell. [file 12935_2024_3271_MOESM1_ESM.zip › New folder/Supplementary Figure 6.png]
